# Mouse blood cells types and aging prediction using penalized Latent Dirichlet Allocation

**DOI:** 10.1186/s12864-024-10763-8

**Published:** 2024-09-18

**Authors:** Xiaotian Wu, Yee Voan Teo, Nicola Neretti, Zhijin Wu

**Affiliations:** 1 Department of Biostatistics, Brown University, Providence, RI USA; 2 Department of Molecular Biology, Cell Biolgy, and Biochemistry, Brown University, Providence, RI USA

**Keywords:** Single cell RNA seq, Penalized LDA, Aging, Blood cells

## Abstract

**Background:**

Aging is a complex, heterogeneous process that has multiple causes. Knowledge on genomic, epigenomic and transcriptomic changes during the aging process shed light on understanding the aging mechanism. A recent breakthrough in biotechnology, single cell RNAseq, is revolutionizing aging study by providing gene expression profile of the entire transcriptome of individual cells. Many interesting information could be inferred from this new type of data with the help of novel computational methods.

**Results:**

In this manuscript a novel statistical method, penalized Latent Dirichlet Allocation (pLDA), is applied to an aging mouse blood scRNA-seq data set. A pipeline is built for cell type and aging prediction. The sequence of models in the pipeline take scRNA-seq expression counts as input, preprocess the data using pLDA and predict the cell type and aging status.

**Conclusions:**

pLDA learns a dimension reduced representation of the expression profile. This representation allows identification of cell types and has predictability of the age of cells.

## Introduction

Single cell RNA sequencing (scRNA-seq) is a recently developed technology that allows the quantification of RNA transcripts at individual cell level. Traditional RNA-seq measures gene expression in “bulk” samples by sequencing RNA molecules pooled from a large number (thousands to millions) of cells. Bulk RNA-seq therefore measures only the average expression in a population of cells, and does not provide detailed information in individual cells. In contrast, scRNA provides higher resolution in gene expression measurements by revealing the variability between cells. Single-cell technology directly measures the transcriptome and helps to identify gene regulatory networks and reveal unique cell types [1]. In an aging animal, cell of various types and their underlying expression profile may change throughout its lifetime. These changes reveal how aging progresses differently in different cells and cell types.

Like most breakthroughs in recent biotechnology, the new opportunities come with challenges in data analysis and modeling. The raw data from scRNA-seq are similar to that in bulk RNAseq. Both are sequencing reads from short fragments of RNA molecules, except that cell-specific barcodes are added to the RNA fragments in scRNA-seq. After these RNA samples are pooled and sequenced, the barcodes reads the same cell to be sorted. The reads are then mapped to a reference transcriptome, and the data are in the form of a count table. Bulk RNAseq usually have only a handful of biological samples, whereas in scRNA-seq, it is common to have hundreds to thousands of samples in methods like SMARTer [2] to tens of thousands of samples in Drop-Seq [3], each representing a single cell. A consequence of sequencing many cells at the same time is that the sequencing depth is lower in individual cells. Another characteristic of scRNA-seq data is the sparsity, or excess of zero counts. Part of this is biological, since the true average expression in a population of cells is only zero when a gene is not expressed in any of these cells, making it much less likely to happen in bulk samples. In a single cell, it is not as surprising that a gene’s transcript is indeed not present. On the other hand, the reduced sequencing depth also lowered the probability to detect genes with very low concentrations. Sparsity and relative lower sequencing depth are especially observed in Drop-Seq data as the method sacrifices sequencing depth for high throughput. Novel statistical methods need to be developed to cope with these problems.

Many new statistical models and methods have been developed, either to address the challenges associated with scRNA-seq in answering familiar questions, such as identifying differential expression [4–8], or new questions, such as constructing pseudotime [9]. In this manuscript we present an approach to identify cell type and age of cells using a penalized Latent Dirichlet Allocation model. We demonstrate that the transcriptome of cells change as the animal ages but the cells age differently in different cell types.

## Method

### Penalized Latent Dirichlet Allocation

We consider cells as documents in the latent dirichlet allocation (LDA) [10] context, with genes equivalent to words as summarized in Table 1. The collection of all genes in a species corresponds to the whole vocabulary in a language. Genes are indexed by $g \in {1, \dots , G}$ where *G* is the total number of genes in the transcriptome. The data from one cell is a vector of gene counts $\textbf{y}=[y_1, \dots , y_G]^T$, where each $y_g$ is the observed transcripts count in the sequencing experiment for gene *g*.

**Table 1:** Comparison between natural language and scRNAseq

English	Example	RNA Sequencing	Example
Words	regression, brain, cell	Genes	TP53, TNF, NFKB1, BRAC1
Topics	statistics, neural science	Topics	protein synthesis, cell division
Documents	research articles, news articles	Samples	RNA sequencing sample

The LDA model is a generative model that assumes a generative process for each cell (document) in a cell population (corpus). Cells could operate *K* (assumed known and fixed) biological processes, each corresponding to a topic. Each topic has a topic-specific gene expression frequency, described in a $K \times G$ matrix $\varvec{\beta }$, where each row $\varvec{\beta }_k$ describes the conditional gene expression frequency under topic *k* and $\sum\nolimits _g \beta _{kg} =1$. A detailed model specification could be found in [10].

Given the gene count matrix $\textbf{Y}_{G \times N}$, we can use LDA to identify latent biological topics and infer the topic frequency for each cell, $\theta _i$. Since *K* is usually much smaller than *G*, the topic by cell matrix $\varvec{\Theta }$ can be seen as a dimension reduced summary of the transcriptional activity in the *N* cells. The topic-level summary for each cell captures a higher-level, more abstract functional activity of cells, compared to individual gene-level counts.

The matrix $\varvec{\Theta }$ can be used in clustering and classification of cells, as well as comparison of functional differences between cell populations. We are interested in identifying the latent biological topics, as well as the topic-specific gene frequencies, which can help us understand the function of these topics. An illustration of the $\varvec{\Theta }$ and $\varvec{\beta }$ is as follow:

In natural language processing, “stop words”, those that used in a similar and high frequency across various topics, are usually removed before model fitting since they offer little value in separating the topics. In scRNAseq data, there are also genes that have a similar $\beta _{kg}$ across topics and data from these genes bring more noise than inferential value in identifying the topics. We developed a method that includes a penalty term on the heterogeneity of $\beta _{kg}$ for any *g* over the *K* topics. A gene with $\beta _{kg}=\beta _{0g}, \forall k$ has the same frequency in all topics, thus would not be useful in inferring the topics, and can be filtered out. We have penalized log likelihood as

$$\begin{aligned} l^*(\alpha ,\beta )= l(\alpha ,\beta ) + \lambda \sum \limits _{k=1}^{K} \sum \limits _{g=1}^{V} \left( \beta _{kg} - \frac{1}{K} \sum \limits _{l=1}^{K} \beta _{lg}\right) ^2 \end{aligned}$$

where $\lambda$ is the tuning parameter, $l(\alpha ,\beta )$ is the likelihood from the original LDA model. Detailed model setup and optimization could be found in [11]. The algorithm is speed optimized and implemented as an R package (https://github.com/wuxiaotiankevin/pLDA).

### Cell type classification and age prediction

We split the cells from all animals, both young and old, into equal sized training and testing sets. The 50/50 random split is done in each cell type such that after the split, there is a balanced representation of all cell types in the training set, including the rare cell types. A cell type is considered rare if it has less than 30 young cells or 30 old cells in the training data. For this data set, DC, MK, Macrophage, Basophil are considered as rare cell types. We consider the major cell types and merge the subtypes for B- and T-cells.

**Fig. 1:**
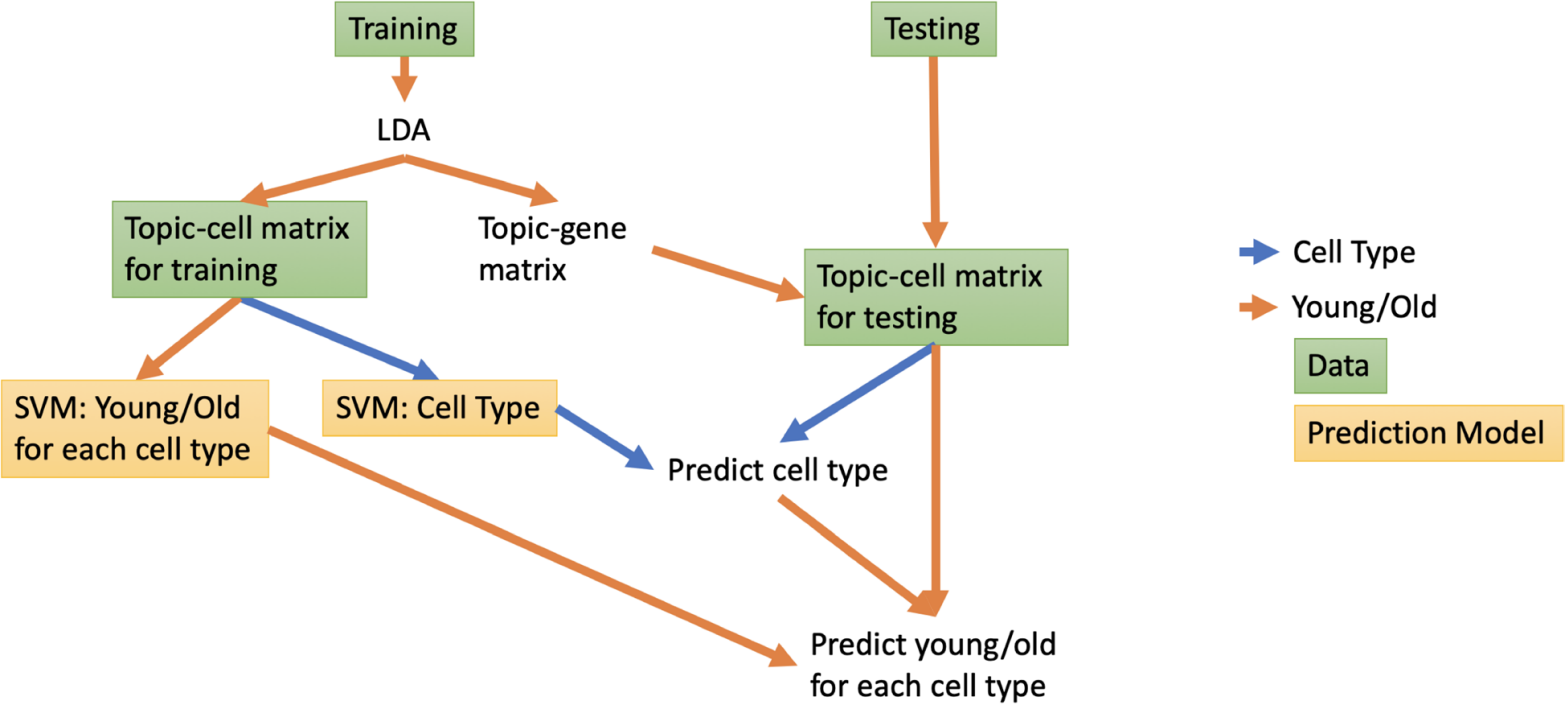
Prediction task setup

We identify the latent topics by fitting the pLDA model on the scRNA-seq data from the training cells. We have previously shown that classification accuracy is not sensitive to the choice of *K* [11]. Here we choose a $K = 17$, the same as the number of cell types. This yields the estimates of $\Theta _{train}$ and $\beta _{train}$. The expression profiles at the topic level are now simplices of length *K*. We use the square root of $\Theta _{train}$ as the input for support vector machine (SVM), for the square-root transformation increases the weight of less abundant topics but does it in a subtler manner compared to the logit transformation. Using the dimension-reduced and transformed $\Theta _{train}$, we fit one SVM to classify cell type. For each cell type, we separately train an SVM to predict the cell age. The procedure is summarized in Fig. 1.

### Gene ontology analysis

We perform gene ontology (GO) analysis for each topic using the “conditional hypergeometric test” in the GOstats package [12] in R with the “org.Mm.eg” annotation package. In the $\beta$ matrix, each gene is represented by a length *K* vector. We only use genes with heterogeneous frequencies across topics in the gene ontology (GO) analysis. Genes with cosine distance smaller than 0.6 to $\vec {1}$ are removed. All the genes left are used as the gene universe. For each gene, a corresponding topic is identified by the largest value in the length *K* vector from the $\beta$ matrix.

For each topic, the enrichment analysis is carried out focusing on the topic specific genes. A p-value of 0.001 is used as the cutoff for calling interesting enriched gene ontology terms.

## Results and discussions

### Mouse aging peripheral blood data

The scRNA-seq data used here are from 14,588 aging peripheral blood cells from 2 young (4 month) and 2 old (24 month) female C57BL/6 mice. The data generation and filtering details are described in [13]. There are 10,361 genes in the data set. 14,588 cells that passed quality filtering are clustered with Seurat (2.3.0) and 17 clusters are identified. Cell types are assigned to the 17 clusters based on general marker genes. These includes 5 subtypes of T cells, 4 subtypes of B cells, 1 cluster of proliferating B or T cells, NK cells, monocytes, dendritic cells, megakaryocytes, macrophage, basophil and red blood cells (RBC). We consider major cell types and combined the subtypes of the B and T cells Cells in the cell type identification analysis. The 14588 cells are randomly divided into training set and testing set, each holding half of all cells with the same cell type composition.

### Gene expression at the topic level

We split the cells from all animals, both young and old, into equal sized training and testing sets. Using the scRNA-seq counts from training cells, we apply pLDA and estimate the latent topics and the topic-level expression profile for each cell.

**Fig. 2:**
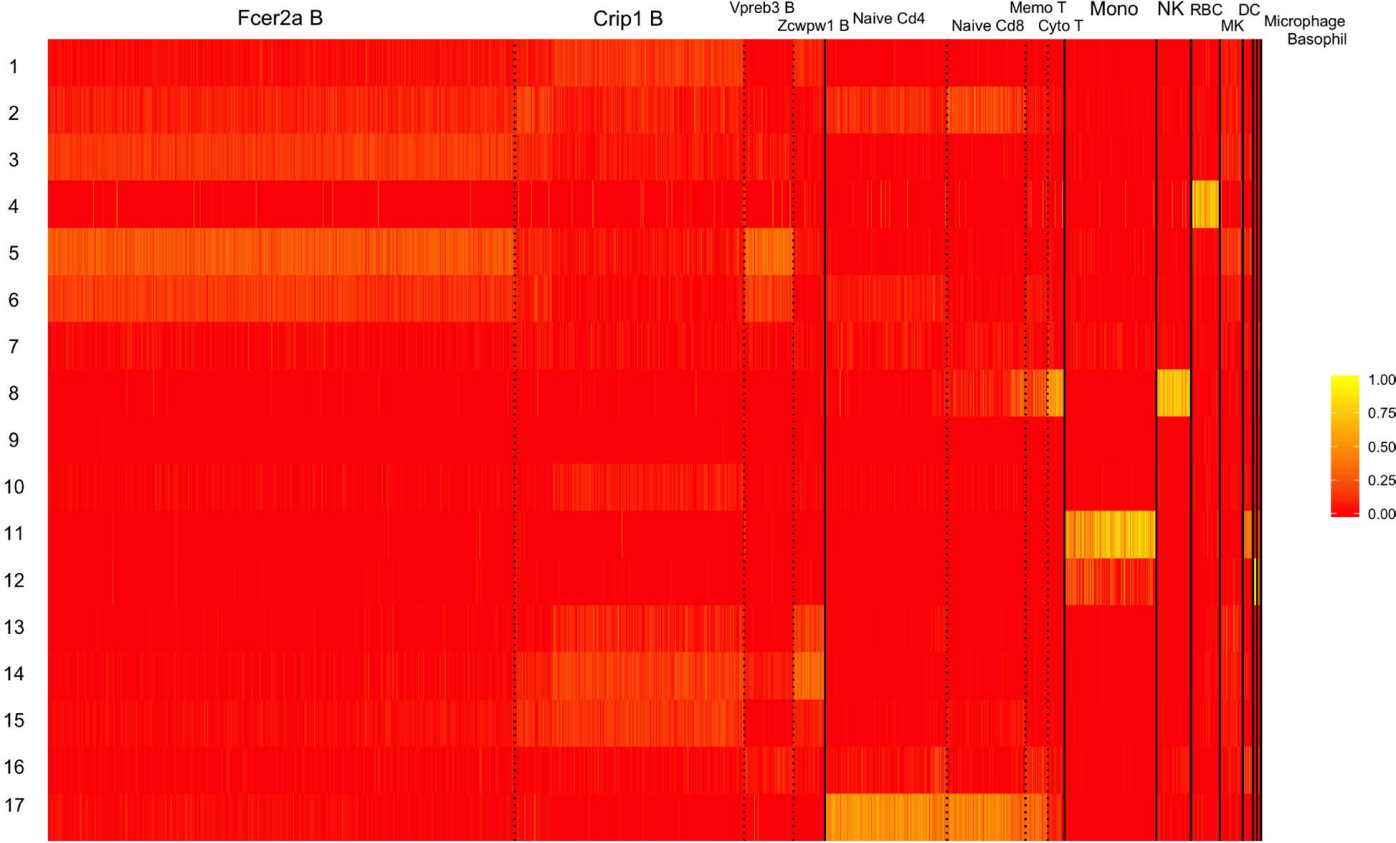
pLDA inferred topic by cell matrix of aging mouse peripheral blood data. Solid black vertical lines divide different cell types

Figure 2 shows the topic-level profiles for the cells in the training set. Each row represents a topic and each column represents a cell. The solid black vertical lines divide different major cell types and dashed black lines divide subtypes. Subtypes in B cells are Fcer2a B, Crip1 B, Vpreb3 B, Zcwpw1 B. Though cell type information is not provided to the pLDA model, and we do not feed any biological gene network to the model, the latent topics inferred from the data lead to a dimension reduced version of the transcriptomes that show distinct patterns across cell types. Some topics are exclusively seen in one cell type. For example, Topic 4 is almost exclusively seen in RBC cells. Topic 17 is mostly observed in T cells, though its activity appears lower in cytotoxic T cells. Other topics are observed in multiple cell types, but with different frequencies. For example, Topic 8 is observed in T cells as well as NK cells. Its frequency is higher in NK cells and Cytotoxic T cells than the other T subtypes.

The estimation process in pLDA does not use cell type information. However, a clear structure is observed for different cell types in the $\Theta$ matrix. This suggests that the topic frequencies obtained from unsupervised learning indeed capture functional information on cell types.

### Cell type classification using topic profiles

**Fig. 3:**
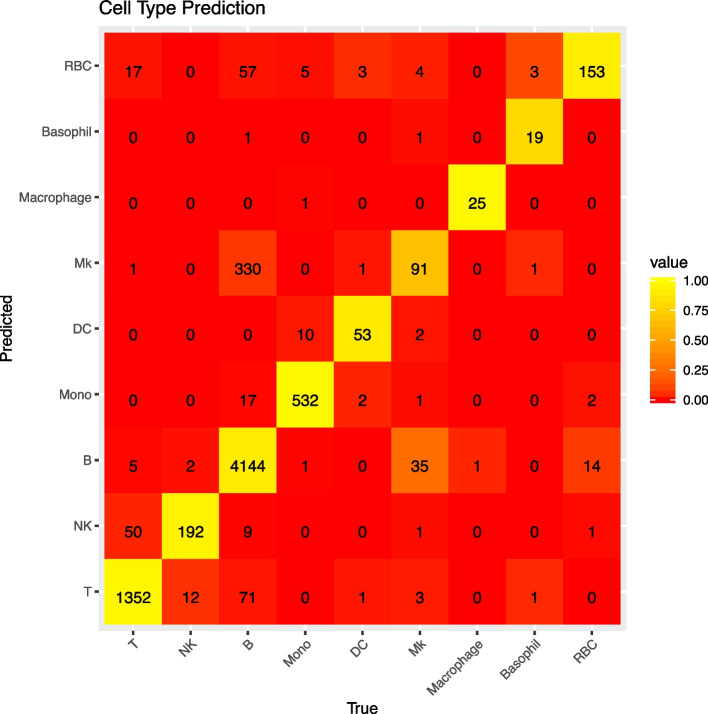
Confusion matrix of cell type classification result on the test data set

We train a SVM using topic profiles from half of the cells as input to identify the major cell types. The expression profiles summarized as biological topic profiles, though in the form that is highly dimension-reduced, reserve most of the information about cell type specific characteristics. Therefore, the SVM built on the topic frequencies accurately recover most cell’s cell type. Figure 3 shows the confusion matrix on the testing cells. The overall accuracy is 91%. These expression profiles are well maintained regardless of age, and the cells are well classified when the age of the animal is not used in our classifier. Though cells from both young and old mice are included, the accurate recall percentage ranges from 79% to 97%, with the exception of MK cells, which are almost exclusively identified in old mice and only recalled with 66% accuracy.

Although the cell type classification is trained using labeled cells from both young and old mice, it appears that young cells are easier to identify. Table 2 shows the recall accuracy of each cell type for cells from old and young mice separately (we do not include the MK cells here as there is only one MK cell detected in the old mice in the test set). In all cell types except NK cells, the accuracy is higher in younger cells, suggesting that cells from old mice may not maintain their identity as well as younger cells.

**Table 2:** Recall accuracy for cell types from different age

	T	NK	B	Mono	DC	Macro	Baso	RBC
Young	.87	.77	.86	.83	.70	.73	.60	.80
Old	.81	.82	.77	.73	.62	.73	.56	.56

**Fig. 4:**
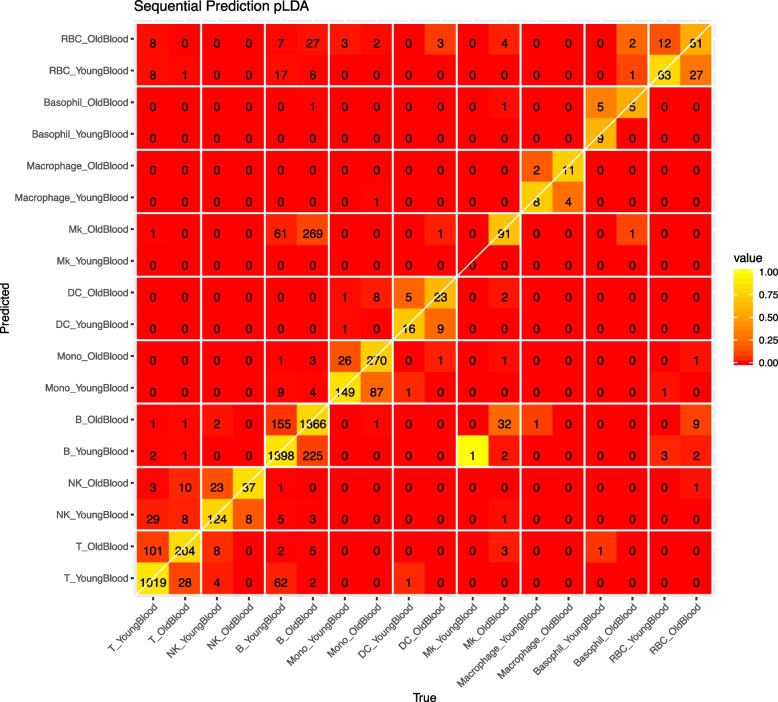
Confusion matrix of cell type and aging prediction using pLDA as dimension reduction on test data set

The most errors in cell type identification appear in B cells, and the error is not entirely random. The B cells that are mis-identified are mostly falsely identified as MK cells. Interestingly, MK cells appear to resemble old B cells (Fig. 4).

### Aging identification

We find that directly making dual predictions of cell type and age does not produce satisfactory result. This is likely due to that cells of different types age differently. We find that a hierarchical approach that first identifies a cell’s cell type, and then identifies its age, produces better accuracy. Figure 4 shows the confusion matrix when we consider age groups in each cell type. Each block consists of a $2\times 2$ table that sum up to the corresponding cell in Fig. 3.

There is clearly age-dependent expression changes in most cell types. The the abundant cell types (T, NK, B, Monocytes), the accuracy of age prediction is high. This is reflected by the higher values on the diagonal, and within each $2\times 2$ block along the diagonal, in Fig. 4. Given the correct cell type, the accuracy of inferring a cell’s age ranges from 74% to 100%, with the higher accuracy in more abundant cell types. The accuracy is at least 79% for cell types that have at least 500 cells in the training data set. The MK cells are rarely detected in young mice, thus the age prediction is not as meaningful for these cells.

### Aging represented as change of biological topic frequency

**Fig. 5:**
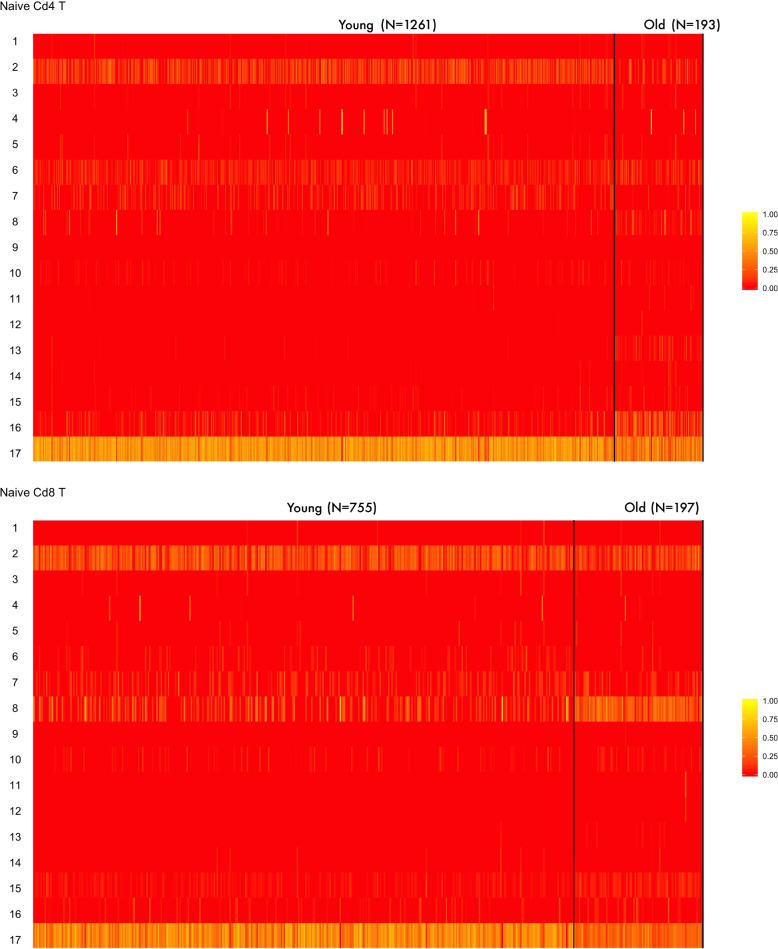
$\Theta$ matrix for naive CD4 T and naive CD8 T comparing young and old

The change in the gene expression in aging is captured in the change of biological topic frequency. Figure 5 shows the topic frequencies between young and old cells in Naive CD4 T cells and Naive CD8 T cells. We can see that most of the cells maintain a high frequency of Topic 17, the marker topic for T cells. This is consistent with the observation that most T cells, regardless of age, can be correctly identified as T cells. However, the topic frequency of Topic 17 is reduced in old cells, suggesting that in older animals the T cell’s most prominent biological function is reduced. We can also see that, compared to young T cells, Topic 16 is more prevalent in old CD4 T cells, whereas Topic 8 is more prevalent in CD8 T cells. In the topic specific gene frequencies section we provide an interpretation of these topics.

**Fig. 6:**
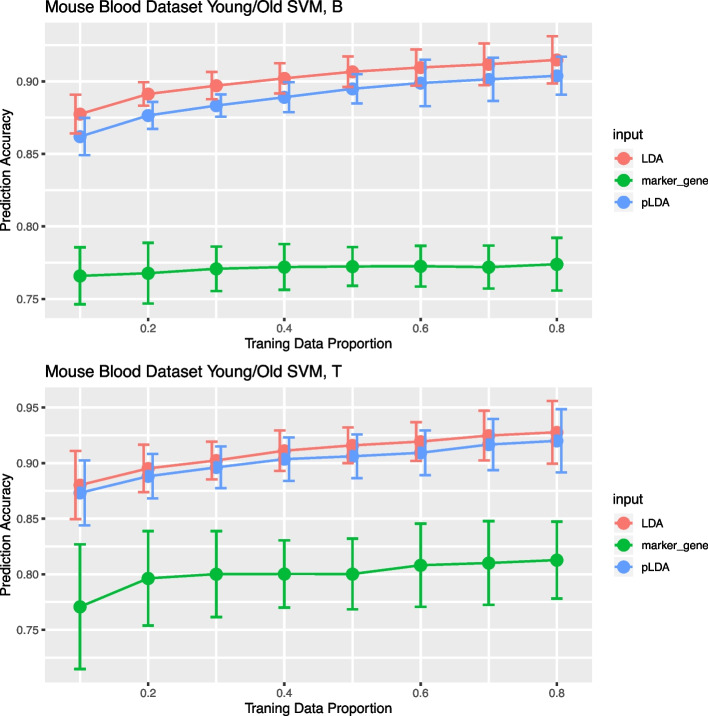
Comparing young/old prediction accuracy for B cells and T cells using pLDA, LDA and marker gene expressions

For the two most abundant cell types, B cells and T cells, we evaluated the prediction accuracy as we increase the amount of training data. The accuracy can be further increases as we use more cells in the training (Fig. 6). Using the topic frequency produces even better prediction for age compared to using the marker genes for these cell types, where the marker genes are the ones identified in [13]. pLDA and LDA provide similar results. Both show a substantial improvement over the marker genes.

### The topic specific gene frequencies

In many natural language processing applications, the focus is put on the dimension reduced profile (the $\Theta$ matrix) and the downstream clustering or classification based on the reduced data. The topic-specific frequency that explains the differences among the topics is often discarded as a byproduct. However, in the scRNA-seq context, the topic-specific gene frequency matrix is a key output as it explains the differences between the biological topics and provides biological interpretation.

**Fig. 7:**
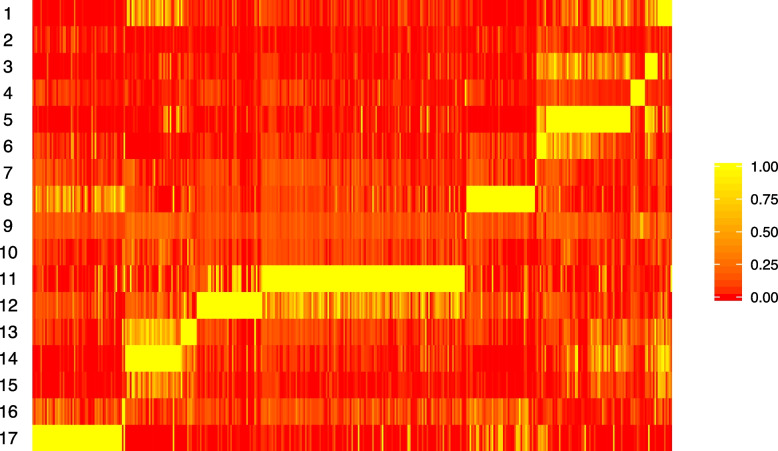
pLDA inferred biological topics from aging mouse peripheral blood data. Each row is gene frequencies in a latent biological topic. Each column represents a gene. Only genes with heterogeneous frequencies across topics are included in this plot. Genes are sorted by the relative frequency across topics

Figure 7 shows the 17 latent topics identified from the pLDA model. This matrix defines the biological functions of each topic. For easier visualization, we rearrange the genes such that ones with high frequency in the same topic are grouped together. Gene ontology (GO) analysis is performed for each topic to identify the enriched biological functions in each topic. The top GO terms significantly enriched by topic are summarized in Table 3. For example, the first 5 rows of Table 3 summarizes the top enriched GO terms for topic 4 learned from pLDA. Topic 4, which is mostly seen in red blood cells (RBC) in Fig. 2, has enriched gene expression in erythrocyte (RBC) differentiation, development and homeostasis. Topic 5 is mostly active in B cells. The corresponding GO terms are related to antigen processing and B cell activation. Topic 8 is mostly observed in natural killer (NK) cells. The gene ontology terms are all natural killer cell related. Topic 17, which is most abundant in T cells, has enriched T cell terms like T cell activation and T cell differentiation. Note that these topics are not supervised or guided by biological knowledge. Instead, these are automatically detected in the pLDA model.

**Table 3:** Mouse blood aging data GO terms

Topic	Term
4	erythrocyte development
4	erythrocyte differentiation
4	erythrocyte homeostasis
4	myeloid cell development
4	myeloid cell homeostasis
5	antigen processing and presentation of peptide antigen via MHC class II
5	antigen processing and presentation of peptide or polysaccharide antigen via MHC class II
5	antigen processing and presentation of exogenous peptide antigen via MHC class II
5	B cell activation
8	natural killer cell chemotaxis
8	regulation of natural killer cell chemotaxis
8	natural killer cell activation
8	natural killer cell mediated immunity
11	myeloid leukocyte mediated immunity
11	regulation of myeloid leukocyte mediated immunity
11	myeloid leukocyte activation
11	regulation of vesicle-mediated transport
11	myeloid cell activation involved in immune response
12	complement activation
12	protein activation cascade
12	long-chain fatty acid transport
12	humoral immune response
14	muscle cell differentiation
14	striated muscle cell differentiation
14	muscle cell development
17	T cell activation
17	T cell differentiation
17	lymphocyte differentiation
17	T cell receptor signaling pathway
17	lymphocyte activation

## Conclusions

We present a pipeline for cell type and age group prediction for mouse blood cells based on a novel statistical method, penalized Latent Dirichlet Allocation [11]. The pLDA method produces two outputs: a dimension reduced expression profile that summarizes each cell’s expression activity as frequencies of a small number of biological topics, and a topic-specific gene frequency matrix that describes how each biological topic uses various genes. We show that the topic-level profiles allows the identification of cell types and has predictability of the age of cells.

The accuracy in predicting the age of the cells vary across cell types. This may simply reflect a difference in the size of training set. For the rarer cell types, it is harder to learn how to classify young cells from old cells.

In our pipeline, we keep the topic-specific gene frequency estimated from the training cells and directly use them in the training cells to decompose the gene counts and obtain the topic profiles of the testing cells. This procedure borrows the transfer learning idea and is one of the advantages of our algorithm. To identify the latent topics, it is necessary to have a diverse population of cells that elicit different biological programs to achieve their functions. Therefore, the latent topics cannot be discovered if we only have a homogeneous population of cells. However, once the topic-specific gene frequency (the $\beta$ matrix) is known, we may decompose individual cell’s gene counts to obtain its topic-level profile.

In this manuscript we have only used several thousand cells in the estimation of $\beta$, and these are only blood cells. It is possible that there are certain biological functions that are not active in any blood cells but other cell types. These will not be identified in the $\beta$ matrix we obtain. However, the topics could be learned from a large data set covering a diverse population of cell types. Once learned, there is no need to re-learn the topics each time when we encounter a new data set. The dimension reduced topic profiles, i.e., the topic-cell matrix $\Theta$ for the new data is obtained using previously trained $\beta$. This solves the problem that the new data set might be small and may not be diverse enough to allow all topics to be well estimated. It also saves computation time. As the scRNAseq community continues to accumulate and share data, we will be able to estimate the $\beta$ matrix with increasing accuracy and precision that enable other users to project their gene level expression profile to topic level profile that is more stable and easier to interpret.

## Data Availability

Single-cell RNA-seq of old and young peripheral blood duplicates are accessible through GEO with the accession number of GSE120505 [13]. The pLDA package is available at https://github.com/wuxiaotiankevin/pLDA.

## Abbreviations

GO: Gene ontology
LDA: Latent Dirichlet Allocation
NK: Natural killer
pLDA: Penalized Latent Dirichlet Allocation
RBC: Red blood cell
scRNA-seq: Single cell RNA sequencing
SVM: Support vector machine
